# Cholesterol, triglycerides, HDL, and nitric oxide as determinants of resting heart rate variability in non-hospitalized mild post-COVID individuals: a cross-sectional study

**DOI:** 10.1186/s12872-025-04523-z

**Published:** 2025-01-31

**Authors:** Lucivalda Viegas de Almeida, Aldair Darlan Santos-de-Araújo, Luís Cláudio Nascimento da Silva, Patricia Martins Santos, Mariana Campos Maia, Victória Pereira Frutuoso, Daniel Santos Rocha, Adriana Sousa Rêgo, Daniela Bassi-Dibai

**Affiliations:** 1 Postgraduate Program in Programs Management and Health Services, Universidade Ceuma, Josué Montello, number 1, São Luís, 65075-120 MA Brazil; 2 Postgraduate Program in Physical Therapy, Department of Physical Therapy, Universidade Federal de São Carlos, São Carlos, SP Brazil; 3 Postgraduate program in Microbial Biology, Universidade Ceuma, São Luís, MA Brazil; 4 Department of Physical Therapy, Universidade CEUMA, São Luís, MA Brazil; 5 Postgraduate program in Physical Education, Universidade Federal do Maranhão, São Luís, MA Brazil; 6 Postgraduate Program in Environment, Universidade Ceuma, São Luís, MA Brazil; 7 Postgraduate program in Dentistry, Universidade Ceuma, São Luís, MA Brazil; 8 Healthy Living for Pandemic Event Protection (HL - PIVOT) Network, Chicago, IL USA

**Keywords:** COVID-19, Heart rate variability, Blood plasma lipids, Nitric oxide

## Abstract

**Background:**

The association between plasma lipids, nitric oxide (NO) and cardiovascular risk has been well documented in the literature, however, the association between these outcomes and heart rate variability (HRV) in COVID-19 remains incipient as there is no scientific evidence that has investigated this outcome.

**Objective:**

Investigate whether metabolic outcomes may be associated with cardiac autonomic behavior arising from short-term HRV variables in non-hospitalized mild post-COVID individuals.

**Methods:**

This is a cross-sectional study. Individuals of both sexes, aged ≥ 18 years, who tested positive for SARS-CoV-2 according to the RT-PCR test, without the need for hospitalization, were included. The HRV was collected in the supine position for at least 10 min for later analysis in the Kubios software. Metabolic outcomes [high density lipoprotein (HDL) (mg/dL), cholesterol (mg/dL), triglycerides (mg/dL) and NO (µmol/L)] were collected through a blood sample.

**Results:**

Seventy-three individuals were included (post-COVID = 32; control = 41). HRV was worse in the post-COVID group when compared to the control group (*p* < 0.05). Cholesterol, HDL, triglycerides and NO showed significant correlations with HRV indices. Regression models indicated that cholesterol and triglycerides, as well as NO, explain up to 30.3% of the variations in certain HRV indices, suggesting an impact of metabolic outcomes on autonomic modulation.

**Conclusion:**

There is a relationship between plasma lipids, NO and HRV in non-hospitalized individuals with mild COVID-19. Metabolic outcomes are associated and explain between 16.6% and 30.30% of certain variables of resting HRV in post-COVID individuals.

**Clinical trial number:**

Not applicable.

## Introduction

The heterogeneous clinical presentation of severe acute respiratory syndrome (SARS-CoV-2) challenged the scientific community to demystify and understand the various manifestations of the pathology, thus enabling effective infectious control measures [1]. Early investigations confirmed that the respiratory system was primarily affected, and initial therapies were aligned with the treatment of respiratory dysfunction. However, it wasn’t long before a range of systemic deleterious effects was discovered, necessitating improved decision-making strategies [2–5].

One area of investigation that remains underexplored is the impairment of the autonomic nervous system (ANS). It is known that the inflammatory nature of SARS-CoV-2 infection disrupts the cholinergic and anti-inflammatory pathways, leading to an imbalance in cytokine release controlled by the parasympathetic branch through afferent vagal stimulation [6–8]. Several mechanisms underlying this imbalance have been discussed, but the findings remain inconclusive, which may partially explain this dysregulation [9]. A simple, non-invasive strategy to assess ANS integrity and screen for dysautonomia is the measurement of heart rate variability (HRV), which is compromised in COVID-19 [10, 11].

Emerging evidence also points to altered autonomic regulation in post-COVID individuals, often manifesting as increased sympathetic dominance and reduced HRV. This autonomic imbalance, particularly characterized by heightened sympathetic activity and diminished parasympathetic modulation, has been identified as a key contributor to the cardiovascular dysregulation observed in survivors of COVID-19 [12, 13]. Furthermore, decreased HRV is closely linked to increased inflammation and oxidative stress, two factors that are often exacerbated in post-COVID patients [14, 15]. Endothelial dysfunction and excessive sympathetic activation are associated with increased free radical production, which impairs NO function and further exacerbates autonomic imbalance [16]. This autonomic dysfunction, coupled with impaired endothelial function, creates a vicious cycle that elevates blood pressure, reduces vasodilation, and increases the risk of atherosclerosis and other cardiovascular problems [17–19].

Furthermore, alterations in lipid metabolism, another hallmark of COVID-19’s long-term effects, can influence the autonomic regulation of heart rate and vascular tone [20–24]. Dyslipidemia, often seen in COVID-19 survivors, contributes to the development of atherosclerosis and endothelial dysfunction, further compounding the adverse effects on HRV and overall cardiovascular health [25, 26]. The relationship between NO, lipid metabolism, HRV, and autonomic regulation forms a complex web of interdependent factors that contribute to the cardiovascular risk observed in post-COVID individuals [12–16]. As NO levels decrease, sympathetic tone increases [27], leading to reduced HRV and worsening endothelial dysfunction. At the same time, dyslipidemia can further disrupt these processes by promoting inflammation and oxidative stress, both of which exacerbate sympathetic dominance and lower HRV [23].

However, the relationship between these outcomes and HRV has shown less consistent results, largely due to the limited research on this subject. Nevertheless, evidence has demonstrated a negative association between them in various populations [23, 28–31]. After reviewing scientific databases to better understand the role of metabolic outcomes in autonomic dysfunction, specifically in HRV, we identified a gap in the literature. Thus, we hypothesized that metabolic outcomes (HDL, cholesterol, triglycerides, NO) may be to relate of the behavior of HRV indices. Thus, the objective of this study is to investigate whether metabolic outcomes may be associated with cardiac autonomic behavior arising from short-term HRV variables in non-hospitalized mild post-COVID individuals.

## Methods

### Study design and ethical considerations

A cross-sectional study reported in accordance with the guidelines of the Strengthening Reporting of Observational Studies in Epidemiology [32]. The research was conducted at the Universidade Ceuma (São Luís, MA, Brazil) after the study procedures were approved by the Research Ethics Committee of the institution (protocol number: 4.179.747) and conducted according to Declaration of Helsinki. All participants were informed of the purpose of the study and informed consent was obtained.

### Participants

Individuals of both sexes, aged ≥ 18 years, who tested positive for SARS-CoV-2 according to the RT-PCR test, without the need for hospitalization composed the post-COVID group. Participants were recruited in the city of São Luís - MA (Brazil) from February 2022 to March 2023. The stratification followed the COVID-19 Treatment Guideline [33] classification produced by the National Institute of Health: mild cases (individuals who presented signs and symptoms of the disease, such as fever, cough, sore throat, malaise, pain, headache, muscle pain, nausea, vomiting, diarrhea, loss of taste and smell, but without the need for additional oxygen therapy and/or mechanical ventilation). Volunteers who had a myocardial infarction (up to six months from the start of data collection), implanted pacemaker or any metal synthesis, history of heart disease, unstable angina, uncontrolled hypertension, uncontrolled diabetes mellitus and/or dependent chronic obstructive pulmonary disease insulin levels, neoplasms, cognitive impairment, declared users of illicit drugs and pregnancy were not included in the study.

The control group was recruited following strict criteria to ensure homogeneity of participants in terms of general health and absence of exposure to COVID-19. Individuals included in the control group were apparently healthy, of both sexes, with no reported comorbidities, and had no history of chronic diseases or conditions that could influence the results of the study. All participants were selected from a single-center, pre-pandemic database of the laboratory where the research group conducting this study is based, using data from prior research [34]. Additionally, participants were interviewed by the research team in the city where the study took place. Information was disseminated verbally, through posters, and via the internet, ensuring that all participants had not been diagnosed with COVID-19 at any time prior. These criteria ensured that the control group served as an adequate comparative basis for evaluating the specific effects of the conditions under study.

### Sample size

The sample size was established using Ene software, version 3.0 (Autonomous University of Barcelona, Spain) and based on a previous correlation study on HRV [35] and others on the correlation of NO with cardiometabolic risk parameters [36]. We calculated the required number of participants with a one-tailed test, an effect size of *r* = 0.50, an alpha level of 5%, and a statistical power of 95% (1 − β = 0.95). The choice of an effect size of *r* = 0.50 was guided by findings from previous studies that reported associations of similar magnitude between the variables of interest. This threshold reflects a moderate effect size, deemed appropriate for detecting meaningful relationships. To account for potential data loss, we anticipated a dropout rate of 30%, which led to an adjusted sample size of 32 participants per group. This ensures the study is adequately powered to detect significant associations. The use of a one-tailed test was justified by the directional hypothesis based on prior evidence.

### Habitual physical activity – baecke questionnaire

It is a self-administered instrument based on self-report and assesses physical activity carried out in the last 12 months. It consists of 16 items and divided into 3 domains: occupational domain (items 1 to 8), sports domain (items 9 to 12) and leisure domain (items 13 to 16). Response scores are ranked according to the Likert Scale (1–5) [37]. The occupational domain score is made by adding the indicated answers and dividing by 8 (for item 2 the indicated value must be subtracted by 6). The sport domain score is calculated by adding the indicated values and dividing by 4. To calculate the leisure domain score, all selected answers must be added together and the value must be divided by 4 (for item 13, the indicated value must be be subtracted by 6). For each domain, the final score varies from 1 to 5; the higher the score, the higher the level of physical activity [37, 38].

### Mini mental state examination (MMSE)

The MMSE, previously validated for the Brazilian version [39], was applied by an experienced examiner in the form of an interview. The test aims to evaluate different cognitive parameters, containing questions that are grouped into 7 categories: temporal orientation (5 points), spatial orientation (5 points), recording of three words (3 points), attention and calculation (5 points), recall of the three words (3 points), language (8 points) and visual constructive capacity (1 point). The MMSE score ranges from 0 (highest degree of cognitive impairment) to 30 points (best cognitive ability).

### R-R intervals recordings – heart rate variability

Before the collection of the biological signal for the analysis of HRV, patients received some instructions 24 h in advance: abstain from alcohol, caffeine, nicotine, chocolate, soft drinks and energy drinks; avoid intense physical exercise; and ensure a restful night’s sleep both the day before and on the day of the examination. To minimize the effects of the circadian rhythm, all assessments were conducted in the morning, in a quiet environment with humidity and temperature levels controlled between 50 and 60% and 20–24ºC, respectively [40, 41]. Patients remained lying in the supine position for approximately 10 min to allow heart rate stabilization after the postural change. Then, the R-R intervals were recorded using the Polar S810i heart rate monitor (Polar Electro, Kempele, Oulu, Finland) and later transferred to a computer using the Polar Advantage software (Kempele, Oulu, Finland) to perform the HRV analyses.

### Analysis and data processing

The Kubios HRV software, developed by the University of Eastern Finland, was used to process heart rate variability (HRV) data. This intuitive program offers a wide range of options for analysis in the overview, linear analysis (time domain and frequency domain), and nonlinear domain [42–44]. The data were examined in stable sessions, each containing 5-minute periods, focusing on short-term HRV analysis [40]. The signal quality was visually assessed to ensure the following criteria were met: (1) absence of large outliers in the R-R intervals; (2) equidistant R-R intervals; and (3) adherence to the Gaussian distribution of RR intervals and heart rate [42, 45]. The series of R-R intervals underwent trend reduction using a smoothing method with a lambda value of 500 and cubic interpolation at a standard rate of 4 Hz [42, 45].

In the overview, the following variables were analyzed: parasympathetic nervous system (PNS): reflecting parasympathetic activity are on average equal to the normal population average; sympathetic nervous system (SNS): reflecting sympathetic activity are on average equal to the normal population average; and stress index: geometric measurement that is related to the stress of the cardiovascular system. High values indicate reduced HRV and high sympathetic cardiac activation.

The following variables from the linear analysis in the time and frequency domains were obtained: mean duration of the R-R interval (Mean RR) in ms; standard deviation of RR intervals (SDNN); average heart rate in beats per minute (Mean HR bpm); square root of successive RR mean square differences (RMSSD) in ms; integral of the density of the interval histogram RR divided by its height (RR Tri); baseline width of a histogram displaying RR intervals (TINN); relative power of the low-frequency (LF) band (0.04–0.15 Hz) in normalized units (n.u.); relative power of the high-frequency (HF) band (0.15–0.4 Hz) in normalized units (n.u.); ratio of LF-to-HF power [42, 43].

Nonlinear analysis of HRV was performed to obtain the standard deviation perpendicular to the line of identity (SD1) in ms (representing parasympathetic modulation); plot the standard deviation along the line of identity (SD2) in ms (representing sympathetic modulation); ratio of SD2/SD1; approximate entropy (ApEn), sample entropy (SampEn); detrended fluctuation analysis, which describes short-term fluctuations (DFA α1); and Detrended fluctuation analysis, which describes long-term fluctuations (DFA α2) [43, 46].

### Metabolic outcomes

Participants were instructed to fast for 8 to 12 h before collection. During this period, water consumption was permitted, but solid foods and other beverages were restricted to avoid interference in the biochemical parameters analyzed. Blood samples were collected with the volunteers positioned in a sitting position, obtained from the antecubital vein and stored in ethylenediaminetetraacetic acid tubes without anticoagulant and with VACUETTE separating gel (Greiner Bio-one, Kremsmuster, Austri). The collected material was sent to the Microbial Pathogenicity Laboratory at CEUMA University. Then, the clotted samples were immediately centrifuged at 1,500 repetitions per minute for 10 min to acquire the serum. Aliquots with approximately 500 µL were prepared in microtubes (KASVI, PR, Brazil) and stored at -80 ºC. To evaluate all biochemical markers, the automatic programmed analyzer LABMAX Plenno (Labtest, MG, Brazil) was used. Serum total cholesterol in milligram per deciliter (mg/dL), high-density lipoprotein (HDL) (mg/dL) and triglyceride (mg/dL) levels were assessed according to the manufacturer’s recommendations and protocol.

To evaluate NO in micromoles per liter (µmol/L), the methodology of Griess et al. [47] and Wang et al. [48] was used. Griess reagent was used, prepared in equal parts of 1% sulfanilamide (reagent A) (w/v) and 0.1% N-1-naphtyl-ethylenediam-ine (reagent B) (w/v), diluted in 2.5% (v/v) phosphoric acid. In a 96-well plate (KASVI, PR, Brazil), 50 µL of serum from the clinical and control groups and 50 µL of Griess reagent were added. The standard curve was created from the microdilution (1:2) of 1 nM/mL sodium nitrite. The microplate was left to rest for 15 min in a dark environment. After resting, the result was read on a spectrophotometer at 535 nm.

### Covariates

The covariates included in the present analysis constitute a broad spectrum of factors associated with HRV and unfavorable outcomes in COVID-19: serum total cholesterol [49], high-density lipoprotein (HDL) [30], triglyceride [50], and NO [51].

### Statistical analysis

Initially, the normality of the data was tested using the Shapiro-Wilk test. Descriptive variables were expressed as mean ± standard deviation (SD). Categorical variables are expressed as frequencies and percentages. For the analysis between the groups (post-COVID group versus control group) T-test for independent samples was used when the data presented a normal distribution (*p* > 0.05). When the data presented a non-parametric distribution (*p* < 0.05), the Mann-Whitney test was used [52]. The categorical variables was compared using χ^2^-square test [53]. Spearman’s or Pearson’s correlation coefficient was used to explore the association between variables depending on the distribution of the data.

We employed univariate and multivariate regression models to analyze the associations between lipid profiles, NO levels, and HRV indices. This approach was chosen because it allows us to adjust for potential confounding factors and evaluate the independent contributions of each metabolic variable to autonomic modulation. Univariate analysis was used to investigate the relationships between HRV and covariates. Control variables with a *p* < 0.20 in the univariate analyses were incorporated into the multivariate model using the stepwise forward method, and those with a *p* < 0.05 in the final model were considered significantly associated with dependent variable [54]. We adjusted the final multiple regression model by the method of inserting variables that improved the explanation of variance: adjusted R^2^. To better understand the direction and magnitude of the relationships between the predictor and dependent variables, the beta value should be interpreted as follows: β (+) suggests a direct relationship, where an increase in the predictor variable leads to an increase in the dependent variable; β (-) suggests an inverse relationship, where an increase in the predictor variable leads to a decrease in the dependent variable.

The homoscedasticity of the residuals was assessed by means of a scatter plot of the unstandardized residuals in relation to the fitted values, which should be randomly distributed and not exhibit discernible patterns. This assessment was performed to verify the assumption that the variance of the residuals is constant across the fitted values ​​and to ensure the homoscedasticity assumption required for regression analysis [55]. We used the Durbin-Watson test to check for the presence of autocorrelation in the residuals in our regression model [56]. It provides a statistic that ranges from 0 to 4: values ​​close to 2 suggest that the residuals do not present significant autocorrelation. Values ​​less than 2 indicate positive autocorrelation, while values ​​greater than 2 indicate negative autocorrelation [56]. Collinearity between independent variables was assessed using the Variance Inflation Factor (VIF) and tolerance: VIF below 10 and tolerance close to 1 were considered acceptable to rule out the presence of collinearity [57]. We considered a number of 15 participants for each independent variable added to the final multiple regression model [55, 58]. To verify the normality of the residuals of the regression model, the Kolmogorov-Smirnov test for standardized residuals was used and a *p*-value > 0.50 was used to accept the hypothesis that the residuals are normally distributed. Cohen’s d was used as a measure of effect size to assess the magnitude of the difference between two groups with the values ​​being interpreted as follows: 0.2: small effect; 0.5: medium effect; 0.8 or higher: large effect. All statistical analyses were performed using the Statistical Package for the Social Sciences (SPSS) 20.0 (IBM, Armonk, New York).

## Results

Seventy-three individuals participated in the study (post-COVID group: *n* = 32; control group: *n* = 41). Table 1 summarizes their personal, anthropometric, and clinical characteristics. The participants were predominantly young adult women (65.6%), overweight based on BMI, and had low levels of habitual physical activity according to the Baecke questionnaire. Obesity (15.6%) was the most common comorbidity in the post-COVID group, followed by systemic arterial hypertension (9.4%) and diabetes (9.4%), with significant differences compared to the control group (*p* < 0.05).

**Table 1:** Personal, anthropometric and clinical characteristics of the included sample (*n* = 73)

Variables	COVID-19 group (*n* = 32)	Control Group (*n* = 41)	*P* value
Age (years)	34 ± 13	34 ± 11	0.971
Gender			
Male	11 (34.40)	22 (46.30)	0.143
Female	21 (65.60)	19 (53.70)	
Weight (kg)	73.56 ± 19.29	71.15 ± 16.73	0.574
Height (m)	1.69 ± 0.11	1.66 ± 0.10	0.876
BMI (kg/m²)	25.39 ± 8.37	25.43 ± 3.84	0.976
Mini Mental State Examination	26 ± 3	27 ± 3	0.901
Level of physical activity	7.35 ± 1.30	7.42 ± 1.38	0.826
Occupational index	2.69 ± 0.69	2.59 ± 0.51	0.512
Sports activity index	2.21 ± 0.65	2.29 ± 0.75	0.690
leisure activity index	2.53 ± 0.76	2.56 ± 0.65	0.875
Comorbidities			
Systemic Arterial Hypertension	3 (9.40)	0 (0)	0.043*
Obesity	5 (15.60)	0 (0)	0.008*
Diabetes Mellitus	3 (9.40)	0 (0)	0.043*
Heart diseases	1 (3.10)	0 (0)	0.249
Infection time (months)	7 ± 3	-	-
Severity			
Mild	32 (100)	-	-
Metabolic Outcomes			
Cholesterol (mg/dL)	132.66 ± 38.03	-	-
Triglycerides (mg/dL)	244.34 ± 175.83	-	-
HDL(mg/dL)	61.03 ± 2.36	-	-
NO (µmol/L)	244.73 ± 151.36	-	-

Data in mean and standard deviation (SD) or in absolute value and percentage (%). Kg: kilos; m: meters; µmol: micromol; L: liters; HDL: High-density lipoprotein

Table 2 compares HRV indices between the groups. Cardiac autonomic modulation, as measured by SNS, stress index, SDNN (ms), LF (n.u.), HF (n.u.), LF/HF, SD2 (ms), SD2/SD1, ApEn, and DFA α1, indicates a poorer sympathovagal balance in the post-COVID group compared to the control group (*p* < 0.05). Effect sizes (Cohen’s d) ranged from 0.516 to 1.367, highlighting moderate to large differences.

**Table 2:** Descriptive and comparative analysis of HRV indices at rest

Variables	COVID-19 Group (*n* = 32)	Control Group (*n* = 41)	*p* value	Cohen d	95%CI
**Overview**					
SNP Index	-1.86 ± 0.89	-0.77 ± 0.85	0.653	1.256	0.751, 1.761
SNS Index	2.03 ± 2.13	1.19 ± 1.06	0.030*	0.520	0.050, 0.990
Stress Index	17.61 ± 9.18	12.74 ± 9.18	0.003*	0.531	0.006, 1.001
**Time Domain**					
Mean RR (ms)	783.33 ± 103.38	801.12 ± 111.13	0.487	0.165	-0.298, 0.628
SDNN (ms)	28.59 ± 14.88	36.50 ± 15.66	0.032*	0.516	0.046, 0.986
Mean HR (bpm)	78.01 ± 11.23	76.19 ± 9.71	0.462	0.175	-0.638, 0.288
RMSSD (ms)	31.29 ± 20.14	33.51 ± 17.74	0.618	0.118	-0.345, 0.581
RR Tri	7.88 ± 4.12	9.43 ± 3.19	0.075	0.428	-0.040, 0.895
TINN	152.19 ± 80.99	191.88 ± 103.45	0.079	0.421	-0.046, 0.888
**Frequency Domain**					
LF (n.u.)	63.43 ± 14.13	46.43 ± 21.63	< 0.001*	0.955	0.468, 1.443
HF (n.u.)	36.50 ± 14.12	54.49 ± 21.64	< 0.001*	1.011	0.520, 1.501
LF/HF	2.27 ± 1.66	1.27 ± 1.18	0.005*	0.680	0.205, 1.156
**Non-linear Analysis**					
SD1 (ms)	22.16 ± 14.26	23.73 ± 12.56	0.619	0.118	-0.345, 0.581
SD2 (ms)	33.44 ± 16.36	45.59 ± 18.89	0.005*	0.681	0.206, 1.157
SD2/SD1	1.77 ± 0.69	2.05 ± 0.48	0.047*	0.482	0.013, 0.951
ApEn	1.09 ± 0.06	1.18 ± 0.07	< 0.001*	1.367	0.855, 1.880
SampEn	1.77 ± 0.23	1.74 ± 0.24	0.551	0.127	-0.590, 0.336
DFA α1	0.95 ± 0.28	1.13 ± 0.21	0.002*	0.741	0.263, 1.218
DFA α2	0.38 ± 0.16	0.43 ± 0.14	0.171	0.335	-0.130, 0.801

SNS: sympathetic nervous system; PNS: parasympathetic nervous system; RR: intervals between one beat and another; HR: heart rate; SDNN: standard deviation of NN intervals; HR: heart rate; RMSSD: root mean squared differences of successive RR intervals; RR Tri: histogram integral of the RR intervals divided by the height of the histogram; TINN: baseline RR interval histogram width; LF: low frequency band; HF: high frequency band; LF/HF: ration between LF and HF; SD1: standard deviation of instantaneous beat-to-beat variability; SD2: long-term standard deviation of continuous RR intervals; SD2/SD1: ratio between SD2 and SD1; ApEn: approximate entropy; SampEn: sample entropy; DFA α1: detrended fluctuation analysis, which describes short-term fluctuations; DFA α2: detrended fluctuation analysis, which describes long-term fluctuations; ms: milliseconds; n.u.: normalized units; bpm: beats per minute

**Fig. 1:**
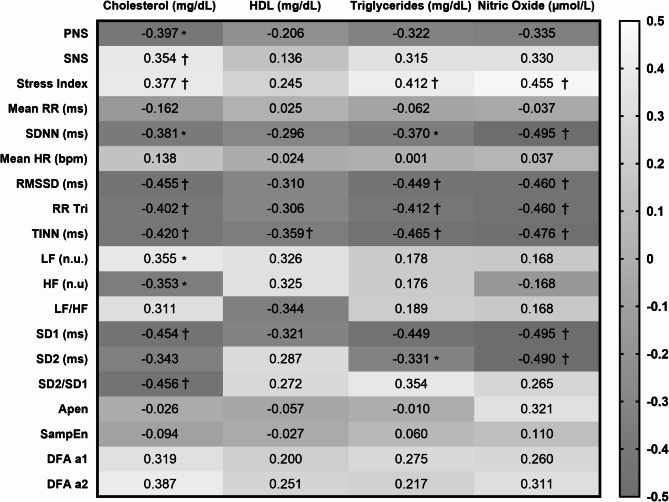
Matrix correlation of HRV variables in the supine position with cholesterol, HDL, triglycerides and NO in post-COVID. HRV: heart rate variability; µmol: micromol; L: liters; ms: milliseconds; n.u.: normalized units; bpm: beats per minute; PNS: parasympathetic nervous system; SNS: sympathetic nervous system; Mean RR: average of R-R intervals; SDNN: standard deviation of RR intervals; Mean HR: average heart rate; RMSSD: root mean square differences of successive RR intervals; RR Tri: integral of the histogram of the RR intervals divided by the height of the histogram; TINN: baseline width of a histogram displaying RR intervals; n.u.: standardized units; LF: low frequency range; HF: high frequency range; SD1: standard deviation of instantaneous beat-to-beat variability; SD2: long-term standard deviation of continuous RR intervals; ApEn: approximate entropy; SampEn: sample entropy; DFA α1: trend fluctuations (short-term scale); DFA α2: trend fluctuations (long-term scale); r: Pearson correlation coefficient; rho: Spearman correlation coefficient; *Significant for Pearson correlation (*p* < 0.05); †Significant for Spearman correlation (*p* < 0.01)

Figure 1 illustrates the correlations between overview variables, linear (time and frequency domain) and non-linear HRV indices, and the outcomes of cholesterol (mg/dL), HDL (mg/dL), triglycerides (mg/dL), and nitric oxide (NO, µmol/L). Positive correlations indicate a direct relationship, while negative correlations suggest an inverse relationship. Cholesterol (mg/dL) showed significant negative correlations (*p* < 0.05) with most HRV indices, including PNS, SDNN (ms), RMSSD (ms), RRTri, TINN (ms), HF (n.u.), SD1 (ms), and SD2/SD1, and positive correlations with SNS, Stress Index, and LF (n.u.). HDL correlated negatively and significantly only with TINN (ms). Triglycerides exhibited negative correlations with SDNN (ms), RMSSD (ms), RRTri, and TINN (ms), while showing a positive correlation with the Stress Index. Finally, nitric oxide (NO, µmol/L) showed significant negative correlations with SDNN (ms), RMSSD (ms), RRTri, TINN (ms), Total Power, SD1 (ms), and SD2 (ms), with a single positive correlation observed with the Stress Index.

Assuming all necessary regression assumptions were met, univariate models were used to examine the relationships between the independent variables (HRV indices) and the dependent variables (metabolic outcomes). Distinct patterns of association between the dependent and independent variables were maintained. As shown in Table 3, cholesterol (mg/dL) and triglycerides (mg/dL) were associated with PNS (β = -0.009 and − 0.0020, respectively; *p* < 0.05) and SNS (β = 0.020 and 0.004, respectively; *p* < 0.05). Additionally, cholesterol (mg/dL) (β = 0.095; *p* < 0.05), triglycerides (mg/dL) (β = 0.021; *p* < 0.05), and HDL (mg/dL) (β = 1.461; *p* < 0.05) influenced the stress index.

**Table 3:** Univariate regression analysis of factors potentially associated with overview parameters of HRV and metabolic outcomes

Variables	Non-standard coefficients		t	*P* value	Adjusted *R*²	Durbin-Watson
	β	Error				
**PNS INDEX** ^a^						
Cholesterol (mg/dL)	-0.009	0.004	-2.373	0.024*	0.130	1.845
Triglycerides (mg/dL)	-0.002	0.001	-2.051	0.050*	0.103	1.895
HDL (mg/dL)	-0.078	0.068	-1.156	0.257	0.011	1.773
NO (µmol/L)	-0.001	0.001	-1.399	0.172	0.030	1.634
**SNS INDEX** ^**b**^						
Cholesterol (mg/dL)	0.020	0.010	2.130	0.041*	0.102	1.756
Triglycerides (mg/dL)	0.004	0.002	1.922	0.065*	0.088	1.717
HDL (mg/dL)	0.272	0.157	1.728	0.094	0.060	1.715
NO (µmol/L)	0.002	0.003	0.874	0.389	-0.008	1.653
**STRESS INDEX** ^**C**^						
Cholesterol (mg/dL)	0.095	0.041	2.338	0.026*	0.126	1.901
Triglycerides (mg/dL)	0.021	0.009	2.298	0.030*	0.133	1.888
HDL (mg/dL)	1.461	0.657	2.223	0.034*	0.113	1.790
NO (µmol/L)	0.014	0.011	1.258	0.218	0.018	1.702

^a^Dependent Variable: PNS Index; ^b^Dependent Variable: SNS Index; ^c^Dependent Variable: Stress Index; Colinearity statistics: tolerance and VIP = 1.000 for all models. *Statistical significance (*p* < 0.05). SNS: sympathetic nervous system; PNS: parasympathetic nervous system; mg/dL: milligram per deciliter; µmol/L: micromole per liter

In the time domain of linear parameters (Table 4), cholesterol (mg/dL), triglycerides(mg/dL) and NO (µmol/L) are determinants in the SDNN (ms) (β = -0.149, β = -0.034; β = -0.039, respectively; *p* < 0.05) and RMSSD (ms) (β = -0.227, β = -0.050; β = -0.050, respectively; *p* < 0.05). Cholesterol (mg/dL) and NO (µmol/L) partially explain the RR Tri variable (β = -0.041 and β = -0.010, respectively; *p* < 0.05). All metabolic outcomes, cholesterol (mg/dL), triglycerides (mg/dL), HDL (mg/dL), and NO (µmol/L) are related to TINN (β = -0.875, β = -0.195, β = -13.963, β = -0.225, respectively; *p* < 0.05). In the frequency domain, only cholesterol (mg/dL) was related to the LF (n.u.) (β = 0.202; *p* < 0.05) and HF (n.u.) (β = -0.201; *p* < 0.05) variables. Cholesterol (mg/dL) and HDL (mg/dL) are related to the LF/HF variable (β = 0.013, β = 0.195, respectively; *p* < 0.05). In the non-linear analysis variables (Table 5), cholesterol (mg/dL), triglycerides (mg/dL) and NO (µmol/L) demonstrated a relationship with the SD1 (ms) (β = -0.161, β = 0.035, β = -0.035, respectively; *p* < 0.05), while SD2 (ms) was related to triglycerides (mg/dL) (β = -0.033; *p* < 0.05) and NO (µmol/L) (β = -0.042; *p* < 0.05). Finally, in the SD2/SD1 and DFA α2 variables, only cholesterol (mg/dL) demonstrated a relationship (β = 0.008; *p* < 0.05).

**Table 4:** Univariate regression analysis of factors potentially associated with linear parameters of HRV and metabolic outcomes

Variables	Non-standard coefficients		t	*P* value	Adjusted *R*²	Durbin-Watson
	β	Error				
**MEAN RR (ms)** ^**a**^						
Cholesterol (mg/dL)	-0.443	0.490	-0.905	0.372	-0.006	1.587
Triglycerides (mg/dL)	-0.063	0.117	-0.540	0.594	-0.026	1.569
HDL (mg/dL)	1.110	7.988	0.139	0.890	-0.033	1.620
NO (µmol/L)	-0.018	0.125	-0.147	0.884	-0.033	1.619
**SDNN (ms)** ^**b**^						
Cholesterol (mg/dL)	-0.149	0.066	-2.258	0.031*	0.117	2.176
Triglycerides (mg/dL)	-0.034	0.014	-2.340	0.027*	0.138	2.473
HDL (mg/dL)	-1.864	1.099	-1.696	0.100	0.057	2.034
NO (µmol/L)	-0.039	0.016	-2.398	0.023*	0.133	1.822
**MEAN HR (bpm)** ^**c**^						
Cholesterol (mg/dL)	0.058	0.053	1.105	0.278	0.007	1.531
Triglycerides (mg/dL)	0.009	0.013	0.726	0.474	-0.017	1.540
HDL (mg/dL)	0.203	0.867	0.234	0.817	-0.031	1.586
NO (µmol/L)	0.001	0.014	0.101	0.920	-0.033	1.575
**RMSSD (ms)** ^**d**^						
Cholesterol (mg/dL)	-0.227	0.087	-2.602	0.014*	0.157	2.116
Triglycerides (mg/dL)	-0.050	0.019	-2.564	0.016*	0.166	2.321
HDL (mg/dL)	-2.480	1.489	-1.665	0.106	0.054	1.892
NO (µmol/L)	-0.050	0.023	-2.201	0.036*	0.110	1.682
**RR TRI** ^**e**^						
Cholesterol (mg/dL)	-0.041	0.018	-2.263	0.031*	0.117	2.193
Triglycerides (mg/dL)	-0.008	0.004	-1.929	0.064	0.089	2.440
HDL (mg/dL)	-0.445	0.308	-1.445	0.159	0.034	1.990
NO (µmol/L)	-0.010	0.005	-2.196	0.036*	0.110	1.765
**TINN** ^**f**^						
Cholesterol (mg/dL)	-0.875	0.354	-2.471	0.019*	0.141	1.730
Triglycerides (mg/dL)	-0.195	0.080	-2.441	0.021*	0.150	2.158
HDL (mg/dL)	-13.963	5.709	-2.446	0.021*	0.138	1.684
NO (µmol/L)	-0.225	0.089	-2.539	0.017*	0.149	1.443
**LF (n.u.)** ^**g**^						
Cholesterol (mg/dL)	0.202	0.097	2.078	0.046*	0.097	2.117
Triglycerides (mg/dL)	0.026	0.023	1.108	0.278	0.008	1.992
HDL (mg/dL)	2.990	1.580	1.892	0.068	0.077	1.855
NO (µmol/L)	0.004	0.026	0.161	0.873	-0.032	2.142
**HF (n.u.)** ^**h**^						
Cholesterol (mg/dL)	-0.201	0.097	-2.069	0.047*	0.096	2.116
Triglycerides (mg/dL)	-0.025	0.023	-1.098	0.282	0.007	1.997
HDL (mg/dL)	-2.977	1.582	-1.882	0.070	0.076	1.858
NO (µmol/L)	-0.004	0.026	-0.159	0.875	-0.032	2.144
**LF/HF** ^**i**^						
Cholesterol (mg/dL)	0.013	0.005	2.567	0.015*	0.152	2.322
Triglycerides (mg/dL)	0.001	0.001	1.014	0.320	0.001	2.120
HDL (mg/dL)	0.195	0.084	2.307	0.028*	0.122	1.959
NO (µmol/L)	0.300	0.001	0.004	0.997	-0.033	2.244

^a^Dependent Variable: Mean RR; ^b^Dependent Variable: SDNN; ^c^Dependent Variable: Mean HR; ^d^Dependent Variable: RMSSD; ^e^Dependent Variable: RR Tri; ^f^Dependent Variable: TINN; ^g^Dependent Variable: LF; ^h^Dependent Variable: HF; ^i^Dependent Variable: LF/HF. Colinearity statistics: tolerance and VIP = 1.000 for all models. *Statistical significance (*p* < 0.05). RR: intervals between one beat and another; HR: heart rate; SDNN: standard deviation of NN intervals; HR: heart rate; RMSSD: root mean squared differences of successive RR intervals; RR Tri: histogram integral of the RR intervals divided by the height of the histogram; TINN: baseline RR interval histogram width; LF: low frequency band; HF: high frequency band; LF/HF: ration between LF and HF; ms: milliseconds; n.u.: normalized units; bpm: beats per minute; mg/dL: milligram per deciliter; µmol/L: micromole per liter

**Table 5:** Univariate regression analysis of factors potentially associated with non-linear parameters of HRV and metabolic outcomes

Variables	Non-standard coefficients		t	*P* value	Adjusted *R*²	Durbin-Watson
	β	Error				
**SD1** ^**a**^						
Cholesterol (mg/dL)	-0.161	0.062	-2.602	0.014*	0.157	2.116
Triglycerides (mg/dL)	-0.035	0.014	-2.564	0.016*	0.166	2.321
HDL (mg/dL)	-1.757	1.055	-1.665	0.106	0.054	1.892
NO (µmol/L)	-0.035	0.016	-2.202	0.036*	0.110	1.6892
**SD2** ^**b**^						
Cholesterol (mg/dL)	-0.148	0.074	-2.000	0.055	0.088	2.206
Triglycerides (mg/dL)	-0.033	0.016	-2.104	0.045*	0.109	2.514
HDL (mg/dL)	-1.990	1.211	-1.643	0.111	0.052	2.124
NO (µmol/L)	-0.043	0.018	-2.393	0.023*	0.132	1.926
**SD2/SD1** ^**c**^						
Cholesterol (mg/dL)	0.008	0.003	2.576	0.015*	0.154	2.611
Triglycerides (mg/dL)	0.001	0.001	2.010	0.055	0.098	2.117
HDL (mg/dL)	0.107	0.050	2.159	0.039	0.106	2.202
NO (µmol/L)	0.000	0.001	0.273	0.786	-0.031	2.325
**ApEn** ^**d**^						
Cholesterol (mg/dL)	-0.011	0.000	-0.143	0.887	-0.033	2.035
Triglycerides (mg/dL)	-0.001	0.000	-0.017	0.986	-0.037	1.890
HDL (mg/dL)	-0.001	0.005	-0.313	0.756	-0.030	2.027
NO (µmol/L)	0.000	0.000	1.653	0.109	0.053	1.877
**SampEn** ^**e**^						
Cholesterol (mg/dL)	− 0.001	0.001	-0.570	0.573	-0.022	1.572
Triglycerides (mg/dL)	-0.001	0.000	-0.167	0.869	-0.036	1.171
HDL (mg/dL)	0.002	0.018	0.133	0.895	-0.033	1.535
NO (µmol/L)	0.000	0.000	0.718	0.478	-0.016	1.587
**DFA α1** ^**f**^						
Cholesterol (mg/dL)	0.002	0.001	1.837	0.076	0.071	2.340
Triglycerides (mg/dL)	0.000	0.000	1.688	0.103	0.062	2.033
HDL (mg/dL)	0.024	0.021	1.120	0.272	0.008	2.114
NO (µmol/L)	0.000	0.000	0.607	0.548	-0.021	2.145
**DFA α2** ^**g**^						
Cholesterol (mg/dL)	0.002	0.001	2.297	0.029*	0.121	1.745
Triglycerides (mg/dL)	0.000	0.000	1.171	0.252	0.013	1.931
HDL (mg/dL)	0.017	0.012	1.418	0.166	0.032	1.908
NO (µmol/L)	0.000	0.000	0.942	0.354	-0.004	1.788

^a^Dependent Variable: SD1; ^b^Dependent Variable: SD2; ^c^Dependent Variable: SD2/SD1; ^d^Dependent Variable: ApEn; ^e^Dependent Variable: SampEn; ^f^Dependent Variable: DFA α1; ^g^Dependent Variable: DFA α2. *Statistical significance (*p* < 0.05). Colinearity statistics: tolerance and VIP = 1.000 for all models. *Statistical significance (*p* < 0.05). SD1: standard deviation perpendicular to the line of identity in ms; SD2: plot the standard deviation along the line of identity in ms; SD2/SD1: ratio of SD2/SD1; ApEn: approximate entropy; SampEn: sample entropy; DFA α1: detrended fluctuation analysis, which describes short-term fluctuations; DFA α2: detrended fluctuation analysis, which describes long-term fluctuations; ms: milliseconds; mg/dL: milligram per deciliter; µmol/L: micromole per liter

The multiple linear regression models (Table 6) demonstrated that: cholesterol (mg/dL) and triglycerides (mg/dL) jointly are related the variables PNS, Stress Index, SDNN (ms), RMSSD (ms), TINN, SD1 (ms), SD2/SD1 (R² adjusted 0.166, 0.183, 0.172, 0.244, 0.204, 0.244, and 0.206, respectively). While cholesterol (mg/dL) and NO jointly are associated SDNN, RMSSD, TINN, SD1 and SD2 (R² adjusted 0.261, 0.279, 0.303, 0.279, and 0.230, respectively).

**Table 6:** Multiple linear regression to investigate the influence metabolic outcomes in HRV

Model	Variables	Non-standard coefficients		t	*P* value	Collinearity statistics		Adjusted R²	ANOVA *p* value	Durbin-Watson
		β	Error			Tolerance	VIF			
**1**	**PNS**									
	Constant	0.378	0.565	0.669	0.509			0.166	0.036*	2.093
	Cholesterol (mg/dL)	-0.007	0.004	-1.744	0.093	0.942	1.062			
	Triglycerides (mg/dL)	-0.001	0.001	-1.644	0.112	0.942	1.062			
**1**	**Stress Index**									
	Constant	4.800	5.805	0.827	0.416			0.183	0.028*	2.110
	Cholesterol (mg/dL)	0.069	0.042	1.634	0.114	0.942	1.062			
	Triglycerides (mg/dL)	0.018	0.009	1.904	0.068	0.942	1.062			
**1**	**SDNN**									
	Constant	46.614	9.049	5.151	0.000			0.172	0.033*	2.759
	Cholesterol (mg/dL)	-0.095	0.066	-1.450	0.159	0.942	1.062			
	Triglycerides (mg/dL)	-0.028	0.014	-1.968	0.060	0.942	1.062			
**2**	Constant	58.257	9.147	6.369	< 0.001			0.261	0.005*	2.261
	Cholesterol	-0.150	0.060	-2.488	0.019	1.000	1.000			
	NO (µmol/L)	-0.040	0.015	-2.616	0.014	1.000	1.000			
**1**	**RMSSD**									
	Constant	61.515	11.853	5.190	< 0.001			0.244	0.010*	2.758
	Cholesterol (mg/dL)	-0.167	0.086	-1.943	0.063	0.942	1.062			
	Triglycerides (mg/dL)	-0.041	0.019	-2.145	0.041	0.942	1.062			
**2**	Constant	73.879	12.221	73.879	< 0.001			0.279	0.003*	2.159
	Cholesterol (mg/dL)	-0.229	0.081	-0.229	0.008	1.000	1.000			
	NO (µmol/L)	-0.050	0.020	-0.050	0.020	1.000	1.000			
**1**	**RR Tri**									
	Constant	15.911	2.572	6.187	< 0.001			0.237	0.007*	2.115
	Cholesterol (mg/dL)	-0.042	0.017	-2.452	0.020	1.000	1.000			
	NO (µmol/L)	-0.010	0.004	-2.391	0.024	1.000	1.000			
**1**	**TINN**									
	Constant	265.729	49.760	5.340	< 0.001			0.204	0.020*	2.415
	Cholesterol (mg/dL)	-0.607	0.362	-1.679	0.105	0.942	1.062			
	Triglycerides (mg/dL)	-0.163	0.080	-2.043	0.051	0.942	1.062			
**2**	Constant	324.534	48.243	6.727	< 0.001			0.303	0.002*	1.941
	Cholesterol (mg/dL)	-0.882	0.319	-2.765	0.010	1.000	1.000			
	NO (µmol/L)	-0.226	0.080	-2.827	0.008	1.000	1.000			
**1**	**SD1**									
	Constant	43.571	8.396	5.190	0.000			0.244	0.010*	2.758
	Cholesterol (mg/dL)	-0.119	0.061	-1.943	0.063	0.942	1.062			
	Triglycerides (mg/dL)	-0.029	0.013	-2.145	0.041	0.942	1.062			
**2**	Constant	52.329	8.656	6.045	< 0.001			0.279	0.003*	2.159
	Cholesterol (mg/dL)	-0.162	0.057	-2.832	0.008	1.000	1.000			
	NO (µmol/L)	-0.035	0.014	-2.467	0.020	1.000	1.000			
**1**	**SD2**									
	Constant	63.848	10.259	6.223	< 0.001			0.230	0.009*	2.301
	Cholesterol (mg/dL)	-0.149	0.068	-2.196	0.036	1.000	1.000			
	NO (µmol/L)	-0.044	0.017	-2.558	0.016	1.000	1.000			
**1**	**SD2/SD1**									
	Constant	0.610	0.440	1.387	0.177			0.206	0.019*	2.517
	Cholesterol (mg/dL)	0.007	0.003	2.164	0.040	0.942	1.062			
	Triglycerides (mg/dL)	0.001	0.001	1.558	0.131	0.942	1.062			

PNS: parasympathetic nervous system; SDNN: standard deviation of NN intervals; HR: heart rate; RMSSD: root mean squared differences of successive RR intervals; RR Tri: histogram integral of the RR intervals divided by the height of the histogram; TINN: baseline RR interval histogram width; SD1: standard deviation perpendicular to the line of identity in ms; SD2: plot the standard deviation along the line of identity in ms; SD2/SD1: ratio of SD2/SD1; mg/dL: milligram per deciliter; µmol/L: micromole per liter. *Statistical significance (*p* < 0.05)

## Discussion

Our main findings are: (1) the HRV indices are significantly correlated with serum levels of plasma lipids and NO. Therefore, we confirm our hypothesis established a priori; (2) metabolic outcomes are related of resting HRV in this population; (3) the results indicate that biochemical variables should be considered in the assessments, understanding and explanation of cardiac autonomic modulation.

It is well-known that lipids, such as cholesterol, triglycerides, and HDL, as well as NO, are metabolic markers that can modulate autonomic function [21, 29, 59, 60]. However, their influence on HRV occurs through complex physiological pathways, including oxidative stress [61], systemic inflammation [62], endothelial dysfunction [63], and activation of the sympathetic nervous system [22]. Elevated levels of cholesterol and triglycerides promote oxidative stress due to increased production of reactive oxygen species (ROS), particularly in vascular tissues [64]. The excess ROS damages autonomic nerve fibers, impairing parasympathetic activity and reducing HRV [65]. At the same time, dyslipidemia is associated with systemic inflammation, characterized by elevated levels of cytokines such as interleukin-6 (IL-6) and tumor necrosis factor-alpha (TNF-α) [66]. These inflammatory mediators disrupt the balance of the autonomic nervous system by intensifying sympathetic stimulation and suppressing parasympathetic modulation [67]. NO, an essential endothelial vasodilator, plays a central role in autonomic regulation [68]. Dyslipidemia reduces the bioavailability of NO by impairing endothelial function, partly due to NO inactivation by ROS [69]. Reduced levels of NO impair baroreceptor sensitivity, a key mechanism in regulating HRV [70]. Furthermore, endothelial dysfunction increases arterial stiffness, making it harder for the heart to adapt to autonomic stimuli [63].

In patients with COVID-19, variables that characterize the lipid profile of this population have been identified as important predictors of unfavorable outcomes, being associated with both severity and mortality, that is, the impairment is independent of severity, and even those with mild cases have altered lipid levels. Therefore, the lipid profile can be used to assess the severity and prognosis of COVID-19 [71–74]. Previously, the association between the lipid profile and cardiac autonomic modulation has already been the subject of investigation. In men with ischemic disease, the RMSSD (-0.38) and SDNN (*r* = -0.43) showed weak to moderate negative correlations with total cholesterol (mg/dL), while in healthy men without ischemic disease group the mean RR intervals (*r* = -0.46), the SDNN (*r* = -0.39) and RMSSD (*r* = -0.38) also presented similar magnitudes of correlations. Interestingly, this last group showed a weak negative correlation (*r* = -0.33) between triglycerides and the mean RR intervals. The authors conclude that hypercholesterolaemia is associated with a decreased 24-h HRV in this population, suggesting an increased risk of sudden cardiac death [30]. In individuals with diabetes mellitus type 2, negative correlations were observed between HRV and total cholesterol indices (RMSSD [*r* = -0.19], HF [*r* = -0.18]). A positive correlation was also observed in the LF/HF ratio (*r* = 0.17). Regarding the association between HDL and HRV indices, no correlation was observed in this study, corroborating the results found in our study [23].

Interestingly, the magnitude and, mainly, the direction of the betas found in both simple and multiple linear regressions converge towards the didactic interpretation of the interaction between HRV and metabolic outcomes. In summary, variables commonly referenced in the literature that reflect global HRV or parasympathetic autonomic modulation, such as PNS, SDNN (ms), RMSSD (ms), HF (n.u.), and SD1 (ms), are inversely associated with metabolic outcomes.

Specifically, higher mean values of cholesterol, triglycerides, and NO are linked to lower parasympathetic autonomic modulation, which, in turn, leads to worse cardiac autonomic modulation. Conversely, variables used to express sympathetic autonomic modulation, such as SNS, LF (n.u.), LF/HF, and SD2 (ms), show a positive relationship, with the beta values indicating that increases in these variables are proportional to the rise in metabolic outcomes. These relationships are not only intuitive but also minimally expected, as scientific literature has long highlighted the cardiovascular risk associated with the metabolic outcomes discussed here [75–78].

Before discussing our results involving NO and HRV, we need to highlight the limitation in the scientific literature on the topic and consider that the association between NO and autonomic control of heart rate remains an unsolved enigma as previously described [79]. The first demonstration of the role of NO (endogenous and exogenous) in cardiac vagal control reflected in HRV and baroreflex sensitivity was recently introduced at the end of the 20th century [51]. What we know that NO interacts closely with the immune [80] and endocrine systems [59]. Blocking NO synthesis has been shown to impair cardiovascular autonomic adaptations induced by physical training [60]. Furthermore, we also know that there is a discussion about the possibility of NO replacement therapy to treat cardiovascular diseases [81], as the attenuation of baroreflex sensitivity and HRV is associated with reduced levels of NO [82].

It has already been demonstrated an interaction between cholesterol, triglycerides, NO, and nervous system [83–85]. For example, we know that the high total cholesterol and triglycerides levels impair NO signaling [86]. Furthermore, there is a discussion about the possibility of converting cholesterol and free radicals into NO [87], as the endothelial NO synthase genetic polymorphisms appear to influence blood cholesterol levels [88]. Therefore, the significant correlations observed between blood plasma lipids, NO, and resting HRV in our study suggest a potential link between these factors in non-hospitalized individuals with mild COVID-19. While these findings align with the existing literature, we recognize that they do not establish causality. The same reasoning applies to the interaction between NO and autonomic function (both in the time domain and frequency domain). These findings should be interpreted with caution and encourage the development of longitudinal studies to clarify the true causal mechanisms behind these associations.

A curious finding is that, in the univariate regression, triglycerides and cholesterol showed statistically significant associations with most of the HRV variables. However, in the multivariate model, where both variables were analyzed simultaneously, neither reached statistical significance, as observed in variables such as PNS, Stress Index, SDNN, and TINN. For the RMSSD and SD1 variables, in the multivariate model, cholesterol did not show statistical significance, while triglycerides remained significant. This result suggests that, although each variable showed an association with the outcome when analyzed in isolation, their effects are relatively weak when both are included in the model, in terms of the variability explained in the dependent variable. In other words, the contribution of each variable is attenuated when analyzed together, possibly due to their interdependence or the way they jointly influence the outcome. This result should be interpreted with caution, as it does not imply that triglycerides and cholesterol are unimportant. In fact, both have biological relevance in the context of HRV. However, in the current model, the lack of statistical significance in the multivariate model may reflect the low magnitude of the individual effect of these variables when adjusted for one another.

It is well known that, even in mild cases of COVID-19, individuals tend to experience a reduction in HRV, which is associated with a higher propensity for adverse cardiovascular outcomes [13]. Although there are no specific studies in the literature directly exploring the relationship between metabolic outcomes, NO, and HRV in individuals with mild COVID-19, the interaction of these markers with post-COVID-19 cardiovascular health can be inferred from previously described mechanisms. For instance, the lipid profile, characterized by elevated LDL and triglyceride levels, as well as reduced HDL levels, has been negatively associated with COVID-19-related mortality in hospitalized patients [89]. Higher serum concentrations of HDL-C have previously been associated with a lower risk of SARS-CoV-2 infection [78]. Additionally, reduced HDL levels in COVID-19 patients have been correlated with an increased risk of developing severe outcomes [90]. Higher HRV is associated with better survival outcomes, particularly in patients aged 70 or older with COVID-19, regardless of major prognostic factors. Conversely, reduced HRV is linked to the need for Intensive Care Unit and admission within the first week of hospitalization [91].

Non-hospitalized individuals with mild COVID-19 who do not have blood plasma lipids, NO, and resting HRV parameters similar to those demonstrated in our study (in mean and standard deviation values), may have other diseases and acute conditions that must be observed quickly. Therefore, we recommend that clinicians and scientists monitor these parameters (biochemical and autonomic) in non-hospitalized individuals with mild Covid-19 who seek primary health care and/or clinical research.

However, it is important to consider that the use of pre-pandemic data for the control group, which may introduce potential biases. The temporal differences between the control and experimental groups could significantly influence the observed results, particularly in the context of HRV. Factors such as physical activity levels, stress, and sleep quality, which are known to affect HRV, may differ between pre-pandemic and pandemic periods, potentially impacting the comparability of the groups.

## Clinical significance and future directions

The findings of this study have important implications for clinical practice, particularly in the context of mild post-COVID individuals. Healthcare professionals should view routine tests not only as tools to monitor metabolic and cardiovascular parameters but also as a way to assess the functioning of the ANS. This approach allows for continuous monitoring of ANS health, helping to screen and to identify early imbalances that could be linked to cardiovascular risks, even in individuals who have experienced mild COVID-19. Integrating these tests into routine screenings can facilitate the early detection of potential issues, enabling timely and personalized interventions. Ultimately, this strategy may help prevent adverse cardiovascular outcomes and improve long-term health outcomes for mild post-COVID individuals.

To enhance the robustness of future research, some improvements in data collection and study design are recommended. First, adopting a longitudinal design could provide a deeper understanding of temporal relationships and causal inferences. Additionally, ensuring a larger and more diverse sample size would improve the generalization of findings across different populations. Future studies are needed to validate these findings and enhance their clinical applicability. Conducting multicenter trials with larger and more diverse populations would strengthen the generalizability of the results. Future research should also consider including participants with varying severities of the disease to better understand how these findings apply across the full spectrum of clinical presentations.

## Limitations

Another The main limitation of the study is the prevalence of comorbidities in the COVID-19 group compared to the control group. However, it is important to emphasize that COVID-19, regardless of its severity, often affects individuals with pre-existing conditions. In addition, the pathology presents different clinical manifestations and severities, and the projection of our results in individuals with moderate, severe, and critical severity should be considered with caution. We were unable to perform a comparative analysis of metabolic outcomes between groups because the control group came from a database of the investigators’ research laboratory and blood collection was not performed. The data from the control group were collected from a pre-pandemic database, which may introduce cohort differences and affect the generalizability of the findings.

## Conclusion

There is a relationship between plasma lipids, NO and resting heart rate variability in non-hospitalized individuals with mild COVID-19. Metabolic outcomes (HDL (mg/dL), triglycerides (mg/dL), cholesterol (mg/dL), and NO) are associated with HRV outcomes and explain between 16.6% and 30.30% of certain variables of resting HRV in post-COVID individuals. However, it should be noted that the use of pre-pandemic data for the control group represents a potential source of bias. To address this limitation, future studies should prioritize the simultaneous recruitment of control and experimental groups to ensure temporal and contextual comparability. Studies with larger sample sizes are needed to validate and expand our findings, as the sample size used is limited.

## Data Availability

The datasets used and/or analyzed during the current study are available from the corresponding author on reasonable request.
